# Integrated proteomic and metabolomic analysis elucidates the effects and mechanisms of Qiziyusi decoction on IVF outcomes in advanced maternal age infertility

**DOI:** 10.3389/fendo.2025.1573206

**Published:** 2025-10-10

**Authors:** Qingmei Jin, Jianyun Zhao, Yingjie Ma, Yi Zhang, Menghan Yan, Jingyan Song, Xianling Cao, Zhengao Sun

**Affiliations:** ^1^ The First Clinical College, Shandong University of Traditional Chinese Medicine, Jinan, Shandong, China; ^2^ The College of Traditional Chinese Medicine, Shandong University of Traditional Chinese Medicine, Jinan, Shandong, China; ^3^ Reproductive Center of Integrated Medicine, The Affiliated Hospital of Shandong University of Traditional Chinese Medicine, Jinan, Shandong, China

**Keywords:** IVF, TCM (traditional Chinese medicine), advanced maternal age, outcome, follicular fluid (FF)

## Abstract

**Background:**

Advanced maternal age (AMA) is associated with increased infertility and poor outcomes of *in vitro* fertilization (IVF), with limited effective treatments available. The traditional Chinese medicine (TCM) formula Qiziyusi decoction (QZYSD) is promising for addressing infertility in older women; however, its effects and mechanisms on IVF outcomes remain poorly understood. This study integrated a prospective cohort study, proteomics, and metabolomics to elucidate the effects and mechanisms by which QZYSD improves IVF outcomes in AMA infertility.

**Methods:**

This prospective cohort study included 87 patients with tubal factor infertility who underwent IVF at the Reproductive and Genetic Center of Shandong University of TCM from April 2019 to October 2020, and stratified according to maternal age into the AMA (≥ 35 and ≤ 41), AMA-QZYSD (≥ 35 and ≤ 41), and young maternal age (YMA; ≥ 21 and ≤ 27) groups. The three groups of patients underwent controlled ovarian hyperstimulation using a short luteal phase protocol. In the AMA-QZYSD group, patients started oral administration of QZYSD from the day of pituitary downregulation until the day of oocyte retrieval, and follicular fluid (FF) was collected from all groups. The effects of QZYSD on improving IVF outcomes in AMA infertility were evaluated primarily by assessing cumulative clinical pregnancy (CCP) and miscarriage (CCM) rates, with secondary endpoints including the duration and dosage of gonadotropin (Gn) use, serum levels of follicle stimulating hormone (FSH), luteinizing Hormone (LH) and estrogen (E2) after pituitary downregulation, serum levels of E2 and progesterone (P) on the day of human chorionic gonadotropin (hCG) administration, endometrial thickness (EMt), number of oocytes retrieved, fertilization and cleavage rates, number of high-quality embryos on day 3, and embryo freezing status. Differential metabolites and proteins in FF were detected using ultra-performance liquid chromatography-tandem mass spectrometry and label-free quantitative proteomics. Correlation analysis was conducted to identify metabolites and proteins with significant correlations, and potential pathways were enriched and constructed using the common pathway analysis function in MetaboAnalyst (version 5.0). Finally, a “core target protein-metabolite-signaling pathway” network diagram was constructed using Cytoscape to further elucidate the mechanisms by which QZYSD improves IVF outcomes in patients with AMA infertility.

**Results:**

The study included 87 patients in the AMA-QZYSD experimental (n = 28), AMA control (n = 28), and YMA (n = 31) groups. The baseline demographic and clinical characteristics, such as maternal and paternal age, antral follicle count, basal serum levels of FSH, and E2 levels, were comparable across the groups. Regarding the primary endpoint, there was a trend toward a higher CCP rate in the AMA-QZYSD group compared to the AMA group. However, this difference was statistically non-significant (53.57% vs. 39.29%, *P* = 0.28), while the CCP rate in the AMA group was significantly lower than in the YMA group (*P* < 0.05). The CCM rates indicated non-significant differences among the three groups (*P* > 0.05). For the secondary endpoint, serum levels of E2 on the day of HCG (2391.57 ± 985.09 versus 1833.39 ± 763.49, P = 0.04), the number of retrieved oocytes (9.18 ± 3.90 versus 7.07 ± 2.92, *P* = 0.04) and high-quality embryos on day 3 (1.86 ± 1.58 versus 1.04 ± 1.20, *P* = 0.05) were slightly higher in the AMA-QZYSD group compared to the AMA control group, but both were lower than the YMA group (*P* < 0.05). There were non-significant differences between the AMA-QZYSD and AMA groups regarding Gn usage days, Gn dosage, serum levels of FSH, LH and E2 after pituitary downregulation, serum levels of P on the day of hCG administration, EMt, IVF 2PN fertilizations, and embryo freezing status (*P* > 0.05). A total of 35 differentially abundant metabolites were identified through metabolomics, and 492 differential proteins were detected using proteomics. The integrated metabolomics and proteomics results suggested that QZYSD may improve IVF outcomes in AMA infertility primarily by regulating the expression of component C8 alpha chain (C8A), carboxypeptidase B2 (CPB2), serum paraoxonase/arylesterase 1 (PON1), immunoglobulin heavy variable 3-9 (IGHV3-9) and pantetheinase (VNN1), as well as influencing the protein digestion and absorption and pantothenate and coenzyme A biosynthesis pathways.

**Conclusion:**

QZYSD in IVF for women with AMA infertility is promising for improving clinical pregnancy rates and overall IVF outcomes, potentially through its effect on the FF microenvironment. However, further research is needed to conduct larger randomized controlled double-blind clinical trials and experimental studies to elucidate the efficacy and mechanisms of QZYSD on IVF success in this population.

## Graphical Abstract

QZYSD intervention mitigates age-related decline in oocyte quality and improves reproductive outcomes in advanced maternal age (AMA) patients. Integrated multi-omics analysis of follicular fluid reveals key proteins (CBA, CPB2, PON1, IGHV3-9, VNN1) and metabolic pathways (pantothenate and CoA biosynthesis) potentially underlying this protective mechanism.

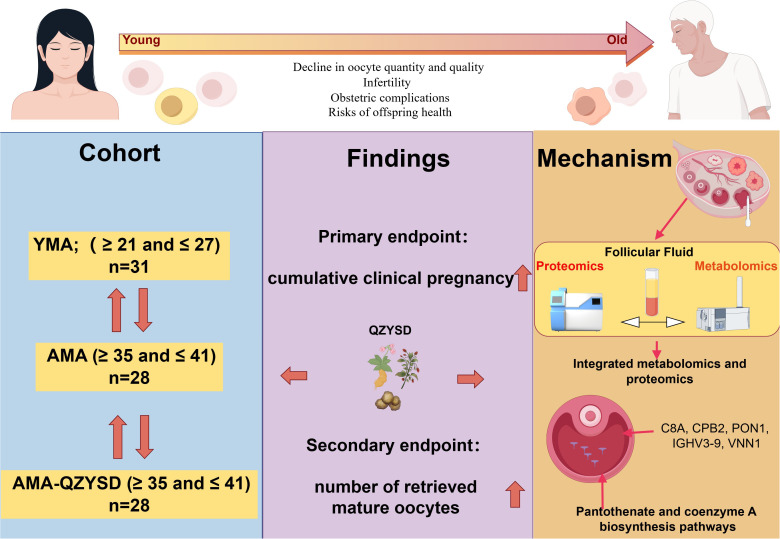

## Introduction

Advanced maternal age (AMA) infertility is a growing concern in modern society, particularly as more individuals and couples delay childbearing for various personal, social, and economic reasons. The prevalence of infertility is estimated to affect 10%–15% of couples globally, with AMA being a significant contributing factor (1, 2). As women age, particularly after 35 years, both the quality and quantity of oocytes decline, resulting in greater challenges in achieving conception (3–6). *In vitro* fertilization (IVF) is an effective approach to address infertility in this population (7); however, the ongoing pursuit of enhancing IVF outcomes for women with AMA remains a significant challenge, drawing considerable attention from researchers aiming to identify effective strategies and interventions.

Traditional Chinese medicine (TCM) offers a multifaceted approach to treating infertility in women with AMA, characterized by its diverse components, multiple targets, low toxicity, and minimal side effects. The qiziyusi decoction (QZYSD), which comprises Rehmannia glutinosa (Shudihuang), Cervus nippon antler powder (Lujiaoshuang), Cistanche deserticola (Roucongrong), Curcuma longa (Yujin), Eclipta prostrata (Mohanlian), Lycium barbarum (Gouqizi), Ligustrum lucidum (Nvzhenzi), Morus alba (Sangshenzi), Rubus idaeus (Fupenzi), Cuscuta chinensis (Tusizi), Nelumbo nucifera (Quanlianzi), Malus pumila (Jinyingzi), and Glycyrrhiza uralensis (Zhigancao), has exhibited considerable promise in improving IVF outcomes for this demographic (8). Recent studies by Zhang et al. have demonstrated that QZYSD significantly enhances endometrial thickness and the number of oocytes retrieved on the day of human chorionic gonadotropin (hCG) administration in patients with AMA (8). Metabolomic analyses of follicular fluid (FF) have identified pathways involving glycine, serine, and threonine metabolism as primary potential targets of QZYSD (8). Additionally, QZYSD is derived from the classical formulation Wuzi Yanzong Pills, which has been demonstrated to alleviate oxidative stress, significantly improve sperm quality, and increase clinical pregnancy rates (9–12). However, the specific effects and mechanisms of QZYSD on IVF outcomes in women with AMA remain unclear and warrant further investigation.

The intricate composition of Chinese medicinal formulations, such as QZYSD, involves numerous pharmacologically active components and multifaceted mechanisms of action, necessitating a comprehensive analysis to elucidate their pharmacological effects. Metabolomics, which examines small-molecule metabolites within biological systems, has gained prominence as a powerful approach to understanding the biochemical pathways influenced by these compounds. Metabolomics enables the identification of potential biomarkers and therapeutic targets by providing insights into metabolic profiles, thereby enhancing our understanding of complex biological interactions. Concurrently, proteomics has emerged as a pivotal discipline in molecular biology, focusing on the systematic analysis of proteins, including their expressions, modifications, and interactions within cellular contexts. This approach offers critical insights into the dynamic processes governing cellular functions and responses to therapeutic intervention. The integration of metabolomics and proteomics, particularly in the context of prospective cohort studies, presents an innovative strategy for dissecting the mechanisms underlying the efficacy of TCM in improving IVF outcomes in women with AMA, ultimately guiding future research and clinical application.

To explore the therapeutic potential of QZYSD in addressing infertility among women with AMA, we used a multifaceted research approach. First, we established a prospective cohort study to evaluate the pharmacodynamic effects of QZYSD on improving IVF outcomes for women with AMA by measuring cumulative clinical pregnancy (CCP) rates, cumulative clinical miscarriage (CCM) rates, and controlled ovarian hyperstimulation conditions. Subsequently, we utilized metabolomics to analyze the metabolic changes in FF and identify the key metabolites associated with treatment response. Proteomics was employed to assess variations in protein expression linked to ovarian function and oocyte quality. We then integrated metabolomics and proteomics data to elucidate the mechanisms by which QZYSD has beneficial effects on IVF outcomes. By validating these findings through further statistical analyses and experimental techniques, we aimed to clarify the functional roles and underlying mechanisms of QZYSD in improving fertility in patients with AMA, ultimately providing potential protein and metabolic pathway targets and guidelines for its clinical application.

## Materials and methods

### Patients and sample collection

This prospective cohort study was approved by the Reproductive Medicine Ethics Committee of the Affiliated Hospital of Shandong University of TCM (approval number: 20190311). All participants provided written informed consent before participating in the research conducted in Jinan, China. From April 2019 to October 2020, 87 patients with tubal factor infertility who received IVF treatment without other female factors in the reproductive center of the Affiliated Hospital of Shandong University of TCM were distributed into different groups according to maternal age: (i) AMA (≥ 35 and ≤ 41, n = 28), (ii) AMA-QZYSD (≥ 35 and ≤ 41, n = 28), and (iii) young maternal age (YMA; ≥ 21 and ≤ 27, n = 31). Patient allocation to the AMA-QZYSD intervention group or the AMA control group was based on a combination of established clinical treatment strategies at our institution and the patients’ fully informed personal preferences after detailed consultation. The definition of AMA was following the 2019 Clinical Practice Guidelines for Assisted Reproductive Technology in Women of Advanced Age in China (13). Inclusion criteria required participants to be between 21 and 41 years old, undergoing IVF solely due to tubal factors, with no history of mental illnesses, and no severe conditions affecting the liver, kidneys, or hematopoietic systems. Additionally, the participants did not use hormonal medications to treat any condition within the three months preceding the study. They were free from endometriosis, polycystic ovary syndrome, premature ovarian failure, ovarian resistance, hyperprolactinemia, thyroid disorders, adrenal dysfunction, or other endocrine diseases. And male oligospermia, asthenospermia, or teratospermia were excluded from the study. Subcutaneous administration of triptorelin, a gonadotropin-releasing hormone agonist (GnRHa; Ferring Pharmaceuticals, Saint-Prex, Switzerland), was initiated at a dosage of 0.05 mg/day starting from the mid-luteal phase of the preceding cycle. This regimen was continued for 14 days to achieve sufficient downregulation of the pituitary gland, defined by follicle-stimulating hormone (FSH) < 5 mIU/mL, luteinizing hormone (LH) < 5 mIU/mL, estradiol (E2) < 50 pg/mL, and endometrial thickness (EMt) < 5 mm. In the AMA-QZYSD group, patients commenced oral QZYSD from the onset of downregulation until the day of hCG injection. The formulation of the QZYSD are outlined in a previously published article (8). The daily dosage of QZYSD provided by the Chinese medicine dispensary of the Affiliated Hospital of Shandong University of TCM was 400 ml, administered in two equal doses of 200 ml each, taken separately half an hour after breakfast and dinner. Follicle development was then stimulated through injections of FSH (Merck Serono SA Aubonne Branch) and human menopausal gonadotropin (Zhuhai Lizhu Group Libao Biochemical Pharmaceutical Co., Ltd.), with dosages tailored based on body weight and follicular response. Once more than three follicles reached a diameter of ≥ 18 mm, a subcutaneous injection of 10,000 U of hCG (Zhuhai Lizhu Group Libao Biochemical Pharmaceutical Co., Ltd) was administered. Oocyte retrieval was performed 35–36 h later via posterior fornix puncture under transvaginal ultrasound guidance. Our primary endpoint was cumulative clinical pregnancy (CCP) from the index retrieval, defined as the presence of an intrauterine gestational sac with cardiac activity after any fresh or subsequent frozen-thawed transfer(s) from that retrieval within the follow-up window. Thus, not only the first transfer, but all transfers belonging to the same retrieval were counted toward CCP. Miscarriage was defined as loss <12 weeks.

Follicular fluid (FF) was collected only from follicles meeting the clinical criteria for oocyte retrieval (typically mean diameter ≥10–12 mm on the retrieval day). All FF aspirates from the same patient were pooled into a single sterile 50-mL conical tube (one tube per patient); no cross-patient pooling was performed. Blood-tinged or turbid aspirates were discarded; only clear, pale-yellow FF associated with MII-confirmed oocytes was retained. Pooled FF was centrifuged at 3,000 g for 15 min at room temperature (Eppendorf 5430R), and the supernatant was transferred to sterile tubes, aliquoted (≈5 mL per cryovial), labeled, and stored at −80°C to preserve protein integrity and avoid repeated freeze–thaw.

### QZYSD preparation and batch quality control

All crude drugs of Qiziyusi Decoction (QZYSD)—Rehmannia glutinosa (Shudihuang), Cervus nippon antler powder (Lujiaoshuang), Cistanche deserticola (Roucongrong), Curcuma longa (Yujin), Eclipta prostrata (Mohanlian), Lycium barbarum (Gouqizi), Ligustrum lucidum (Nvzhenzi), Morus alba (Sangshenzi), Rubus idaeus (Fupenzi), Cuscuta chinensis (Tusizi), Nelumbo nucifera seed (Quanlianzi), Malus pumila (Jinyingzi), and Glycyrrhiza uralensis (Zhigancao)—were supplied and prepared by the Chinese medicine dispensary of the Affiliated Hospital of Shandong University of TCM. Each crude drug lot underwent macroscopic/microscopic authentication and thin-layer chromatography (TLC) identification in accordance with the Chinese Pharmacopoeia (ChP, 2020 edition). Pharmacopoeial safety tests (heavy metals, pesticide residues, aflatoxins, and microbial limits) were performed and met ChP acceptance criteria before use. For each patient-day, weighed crude drugs were combined and soaked in 8–10 volumes of purified water for 30 min, then decocted twice (first 60 min, second 45 min). The combined decoctions were filtered (≈80-mesh) and concentrated under reduced pressure to yield a daily dose of 400 mL, which was hot-filled into two 200 mL sachets. Patients were instructed to take 200 mL twice daily, ~30 min after breakfast and dinner (as reported in this study). Prepared sachets were stored at 4 °C and dispensed every 2–3 days; appearance, pH, relative density (25 °C), and dry-extract content were recorded for in-process control.

During the in-hospital (extemporaneous) preparation phase, our QC standards included unified herb provenance, a standardized decoction procedure, and fixed crude-drug amounts; after obtaining the Shandong Provincial “new drug” approval, we will adopt stricter, GMP-aligned QC and convert the dosage form from decoction to granules, including the following method: For each production batch, we established a UPLC/HPLC chemical fingerprint (DAD at 254/280 nm) against a pooled reference. Fingerprint similarity was evaluated with the Similarity Evaluation System for Chromatographic Fingerprint of TCM (Version 2012), with an acceptance criterion ≥ 0.90. To ensure pharmacological relevance, marker compounds were quantified by external-standard calibration using certified reference standards (National Institutes for Food and Drug Control): acteoside (echinacoside/verbascoside) from Cistanche deserticola, curcumin from Curcuma longa, wedelolactone from Eclipta prostrata, and glycyrrhizic acid (± liquiritin) from Glycyrrhiza uralensis. Marker contents for patient-use batches were required to fall within ±15% of the reference batch, and the RSD of relative peak areas for ≥10 characteristic peaks ≤ 10% across consecutive batches. Short-term stability at 4°C to 72 h was verified by fingerprint similarity and microbial limits (TAMC/TYMC) remaining within acceptance criteria. All batches used in this study fulfilled the above specifications.

### Embryo culture and transfer

Embryos were maintained in a sequential culture system (cleavage medium from day 1–3, then blastocyst medium from day 3–5/6) in 25–30 µL micro-drops under sterile mineral oil, with group culture ≤5 embryos per drop. Media were refreshed at day 3 when moving embryos from cleavage to blastocyst medium. Daily assessments were documented: cleavage-stage morphology (blastomere number, symmetry, fragmentation) was graded per the Istanbul consensus, and blastocysts were graded by the Gardner & Schoolcraft system (expansion stage and inner-cell-mass/trophectoderm quality). Our policy favored blastocyst transfer on day 5 when adequate blastocyst development occurred. Day-3 (cleavage-stage) transfer was performed when blastocyst culture was not advisable (e.g., limited embryo numbers or suboptimal development by day 3). All transfers were performed under ultrasound guidance with a soft catheter. Embryos not transferred were vitrified on day 5/6 according to routine lab SOPs. Importantly, both study arms followed the same laboratory protocol and transfer-day criteria.

### Endpoint

The primary endpoints were the CCP and CCM (miscarriage before 3 months) rates, with secondary endpoints including the number of days and dosage of Gn, serum levels of FSH, LH and E2 after pituitary downregulation, serum levels of E2 and P on the day of hCG, endometrial thickness on the day of hCG, number of oocytes retrieved, number of IVF 2PN fertilization, number of D3 high-quality embryos, embryo freezing rate. Follow-up visits were conducted in the outpatient department on days 14, 21, and 35 and after delivery (if pregnant) following embryo transfer. Given the broad range of targets for TCM, potential beneficial or adverse reactions in the patients were recorded at each follow-up visit. The participants were free to withdraw from the study at any point. The participants self-reported, and spontaneous adverse events associated with QZYSD were documented at each visit from baseline to week 7 of pregnancy through non-guided questioning. Adverse reactions were classified based on severity as follows: Mild symptoms detectable by the patient but tolerable without impacting daily activities or pregnancy continuation; moderate-uncomfortable symptoms affecting the patient’s diet, potentially leading to threatened abortion; and severe symptoms that compromise the patient’s life and health, resulting in pregnancy termination.

### Protein extraction and LC-MS/MS analysis

The FF samples were lysed, and proteins were extracted using SDT buffer (4% SDS, 100 mM Tris-HCl, 1 mM DTT, pH 7.6). The protein concentration was quantified with the BCA Protein Assay Kit (Bio-Rad, USA). Trypsin digestion of proteins was performed following the filter-aided sample preparation protocol established by Matthias Mann (14). The digested peptides from each sample were desalted using C18 cartridges (Empore™ SPE Cartridges C18, standard density, bed I.D. 7 mm, volume 3 mL, Sigma), concentrated by vacuum centrifugation, and reconstituted in 40 μL of 0.1% (v/v) formic acid. LC-MS/MS analysis was conducted using a Q Exactive mass spectrometer (Thermo Scientific) coupled with an Easy nLC (Proxeon Biosystems, now Thermo Fisher Scientific) for 60 min. Detailed parameters and protocols are listed in **Supplementary Table S1**.

### Metabolite extractions and LC-MS analysis

Metabolites from FF samples were extracted by mixing 80 mg of each sample with 1 mL of cold extraction solvent (methanol/acetonitrile/H_2_O, 2:2:1, v/v/v), followed by vortexing, homogenization, and sonication at 4 °C. After centrifugation at 14,000 *g* for 20 min, the supernatant was dried using a vacuum centrifuge and re-dissolved in 100 µL of acetonitrile/water (1:1, v/v) for LC-MS analysis. The analysis utilized a Sciex TripleTOF 6600 mass spectrometer coupled with hydrophilic interaction chromatography, employing an ACQUIY UPLC BEH Amide column. A gradient of solvent A (25 mM ammonium acetate and 25 mM ammonium hydroxide in water) and solvent B (acetonitrile) was applied at a flow rate of 0.5 mL/min and a column temperature of 25°C. The mass spectrometer was operated in negative and positive modes, acquiring data in the m/z ranges of 60–1000 Da for MS and 25–1000 Da for MS/MS using information-dependent acquisition with specified collision energy and declustering potential settings. The detailed methodologies are outlined in a previously published article (15).

### Bioinformatics analysis

Bioinformatics analysis of the proteomics data was conducted using various software tools to elucidate the characteristics and functions of the peptides. Hierarchical clustering of peptides was performed using Cluster (version 3.0) and Java Treeview software, employing the Euclidean distance algorithm for similarity measurement and average linkage clustering. Motif analysis was conducted using MeMe to identify motifs from the extracted amino acid sequences containing modified sites and adjacent residues. For subcellular localization predictions, the CELLO multiclass SVM classification system was used. Domain annotations were obtained using InterProScan to identify the protein domain signatures from the Pfam database. Kyoto encyclopedia of genes and genomes (KEGG, http://www.kegg.jp/) annotations were retrieved by blasting studied proteins against the KEGG database and subsequently mapped to metabolic pathways. Enrichment analysis was conducted based on Fisher’s exact test with Benjamini–Hochberg correction for multiple testing, focusing on functional categories with *P*-values < 0.05. Finally, protein-protein interactions (PPIs) were assessed using data from the IntAct molecular interaction database and STRING software, visualizing interaction networks in Cytoscape and calculating the degree of each protein to evaluate their significance within the PPI network. The detailed methods are described in a previously published article (15).

The metabolomic raw mass spectrometry data (wiff.scan files) were converted to the MzXML format using ProteoWizard MSConvert and then analyzed using XCMS. For peak picking, the parameters were centWave m/z = 25 ppm, peak width = c (10 and 60), and prefilter = c (10 and 100). Peak grouping utilized bw = 5, mzwid = 0.025, and minfrac = 0.5, retaining only variables with over 50% non-zero measurements in at least one group. Metabolite identification involved matching the MS/MS spectra to an in-house database of authentic standards. After normalization to the total peak intensity, the data were analyzed using SIMCA-P (version 14.1, Umetrics, Umea, Sweden) for multivariate analyses, including principal component analysis (PCA) and OPLS-DA. Model robustness was assessed via 7-fold cross-validation and response permutation testing. Variable importance for classification was determined by calculating VIP values in the OPLS-DA model, with statistical significance defined as VIP > 1 and *P* < 0.05.

### Integrated network analysis of the proteome and metabolome

The PCA was conducted using SIMCA (version 14.1) with quantitative data from both omics. Differentially expressed proteins, peptides, and metabolites were mapped to pathways using KEGG, and pathway enrichment analysis was performed. The KEGG annotation and enrichment results from both omics were integrated using R (version 3.5.1), and Venn diagrams and bar plots were generated. The expression profiles of differentially peptides in significantly enriched KEGG pathways were normalized using Z-scores, and heatmaps were constructed using hierarchical clustering (complete linkage and Euclidean distance). Differentially abundant proteins, peptides, and metabolites were scaled using Z-scores (label-free) and combined into a single matrix. Spearman correlation network analysis was performed on proteins and metabolites using R (version 3.5.1) with significant correlation coefficient values |r| ≥ 0.5 and *P* < 0.01. The correlations between differentially expressed proteins, peptides, and metabolites were visualized in CytoScape (version 3.5.1), and a correlation network was constructed. Multivariate statistical analysis of the differentially abundant proteins, peptides, and metabolites was performed with SIMCA (version 14.1).

### Data analysis

The data are presented as mean ± standard deviation for continuous variables with a normal distribution and as n (%) for count data. Statistical analyses were conducted using GraphPad Prism software (version 9). An independent samples t-test or Fisher’s exact test was used to compare groups, as appropriate. In contrast, a one-way analysis of variance was used to analyze differences across multiple groups. *P*-values were adjusted using the Benjamani–Hochberg false discovery rate (FDR), with an FDR ≤ 0.01 considered significant. A *P*-value < 0.05 was considered statistically significant. Receiver operating characteristic (ROC) curves were generated using a 10-fold cross-validation strategy, and model performance was assessed by calculating the area under the curve (AUC) with corresponding 95% confidence intervals.

## Results

### Baseline characteristics

This prospective cohort study included patients with tubal factor infertility who underwent IVF at the Reproductive and Genetic Center of Shandong University of TCM from April 2019 to October 2020. Finally, 87 participants who met the inclusion criteria presented a non-significant difference in baseline data among the AMA-QZYSD and AMA groups in maternal age, paternal age, body mass index (BMI), infertility duration, antral follicle count, and basal hormone levels of FSH, LH, and E2 (*P* > 0.05) (**Table 1**). As expected, no differences were observed in BMI, infertility duration, and basal hormone levels of LH between the YMA, AMA, and AMA-QZYSD groups (*P* > 0.05) (**Table 1**). In contrast, significant differences were found in other parameters among the three groups (*P* < 0.05).

**Table 1:** Baseline characteristics.

Characteristics	AMA-QZYSD(n=28)	AMA (n=28)	*P* value^a^	YMA (n=31)	*P* value^b^
Mean ± SD maternal age (years)	37.18 ± 2.14	38.39 ± 2.18	0.18	25.90 ± 1.83	0.01
Mean ± SD paternal age (years)	37.04 ± 4.71	37.50 ± 4.75	0.63	27.52 ± 2.63	0.01
BMI (Kg/m^2^)	24.40 ± 3.25	23.25 ± 2.99	0.17	22.87 ± 3.13	0.16
Infertility duration (years)	3.32 ± 1.95	3.07 ± 1.94	0.64	2.77 ± 1.45	0.69
No. of AFC (n)	13.07 ± 3.59	12.75 ± 3.05	0.81	19.23 ± 2.93	0.01
Mean ± SD basal hormone levels					
FSH (IU/L)	9.57 ± 1.40	9.45 ± 1.20	0.98	6.70 ± 1.94	0.01
LH (IU/L)	3.72 ± 1.07	3.85 ± 0.97	0.95	4.42 ± 1.60	0.08
E2 (pg/ml)	50.59 ± 9.89	50.30 ± 10.51	0.75	32.58 ± 9.69	0.01

SD, standard deviation; BMI, body mass index; FET, frozen-thawed embryo transfer; No,number; LH, luteinizing Hormone; FSH, follicle stimulating hormone; AFC, antral follicle count; AMA-QZYSD, advanced maternal age qiziyusi decoction; YMA, young maternal age; a, AMA-QZYSD versus AMA, using a chi-square Fisher exact test; b, comparison between groups AMA-QZYSD, AMA and YMA, using a one-way ANOVA test.

### Efficacy of QZYSD on AMA infertility IVF outcomes

Clinical pregnancy rate (%) = number of clinical pregnancies/number of patients × 100%, clinical miscarriage rates (%) = number of abortions/numbers of clinical pregnancies × 100%, the high-quality D3 embryo rate refers to the proportion of high-quality day 3 embryos relative to the total number of normally cleaved embryo. The outcomes are presented in **Table 2**, with data analysis beginning after pituitary downregulation, on the day of hCG administration, and at the other outcome measurement points. The results displayed that the hormone levels of E2 (2391.57 ± 985.09 versus 1833.39 ± 763.49, *P* = 0.04) on the day of hCG, the number of retrieved oocytes (9.18 ± 3.90 versus 7.07 ± 2.92, *P* = 0.04) and the number of high-quality embryos on day 3 (1.86 ± 1.58 versus 1.04 ± 1.20, *P* = 0.05) was higher in the AMA-QZYSD compared to the AMA group (**Table 2**). Although the rate of CCM (3.57% versus 10.71%, *P* = 0.61) and CCP (53.57% versus 39.29%, *P* = 0.28) in the AMA-QZYSD group did not exhibit a statistically significant difference compared to the AMA group, a discernible trend towards a lower CCM rate and a higher CCP rate in the AMA-QZYSD group was observed (**Table 2**). However, there was a statistically non-significant difference in hormone levels of FSH, LH, E2, days of Gn, and total Gn after pituitary downregulation (*P* > 0.05). Besides, the hormone levels of P and EMt on the day of hCG, the number of IVF 2PN fertilization, and embryo freezing rate also had non-significant differences (*P* > 0.05) (**Table 2**).

**Table 2:** The COH and pregnancy outcome.

Characteristics	AMA-QZYSD(n=28)	AMA (n=28)	*P* value^a^	YMA (n=31)	*P* value^b^
Mean (SD) hormone levels after pituitary downregulation					
FSH (IU/L)	4.61 ± 1.09	4.75 ± 1.29	0.80	4.11 ± 1.23	0.07
LH (IU/L)	2.36 ± 1.12	2.08 ± 1.13	0.28	2.00 ± 1.19	0.24
E2 (pg/ml)	32.00 ± 14.61	27.18 ± 12.46	0.16	28.00 ± 18.33	0.32
Days of Gn (U)	10.93 ± 1.49	11.64 ± 1.52	0.07	10.03 ± 1.38	0.01
Total Gn (U)	2630.36 ± 508.44	2745.54 ± 411.47	0.22	1912.50 ± 569.62	0.01
Mean ± SD hormone levels on the day of HCG					
E2 (pg/ml)	2391.57 ± 985.09	1833.39 ± 763.49	0.04	2958.74 ± 841.49	0.01
P (ng/ml)	1.06 ± 0.42	1.07 ± 0.59	0.93	1.21 ± 0.67	0.74
EMt (mm)	11.60 ± 2.63	11.09 ± 2.47	0.45	11.91 ± 2.43	0.46
No. of retrieved oocytes (n)	9.18 ± 3.90	7.07 ± 2.92	0.04	13.32 ± 3.57	0.01
No. of IVF 2PN fertilizations (n)	5.81 ± 3.93	5.65 ± 3.75	0.86	8.04 ± 3.97	0.60
No. of high-quality embryos on Day 3 (n)	1.86 ± 1.58	1.04 ± 1.20	0.05	2.84 ± 1.59	0.01
Embryo freezing rate (%)	39.29%(11/28)	46.43%(13/28)	0.59	67.74%(21/31)	0.07
Cumulative clinical pregnancy rates	53.57%(15/28)	39.29%(11/28)	0.28	74.19%(23/31)	0.03
Cumulative clinical miscarriage rates	3.57%(1/28)	10.71%(3/28)	0.61	12.90%(4/31)	0.52

COH, controlled ovarian stimulation; SD, standard deviation; FET, frozen-thawed embryo transfer; No, number; E2, estradiol; P, progesterone; LH, luteinizing Hormone; FSH, follicle stimulating hormone; Gn, gonadotropin; EMt, endometrial thickness; AMA-QZYSD, advanced maternal age qiziyusi decoction; YMA, young maternal age; a, AMA-QZYSD versus AMA, using a chi-square Fisher exact test; b, comparison between groups AMA-QZYSD, AMA and YMA, using a one-way ANOVA test.

### Identification of differentially expressed proteins and metabolites

**Figure 1:**
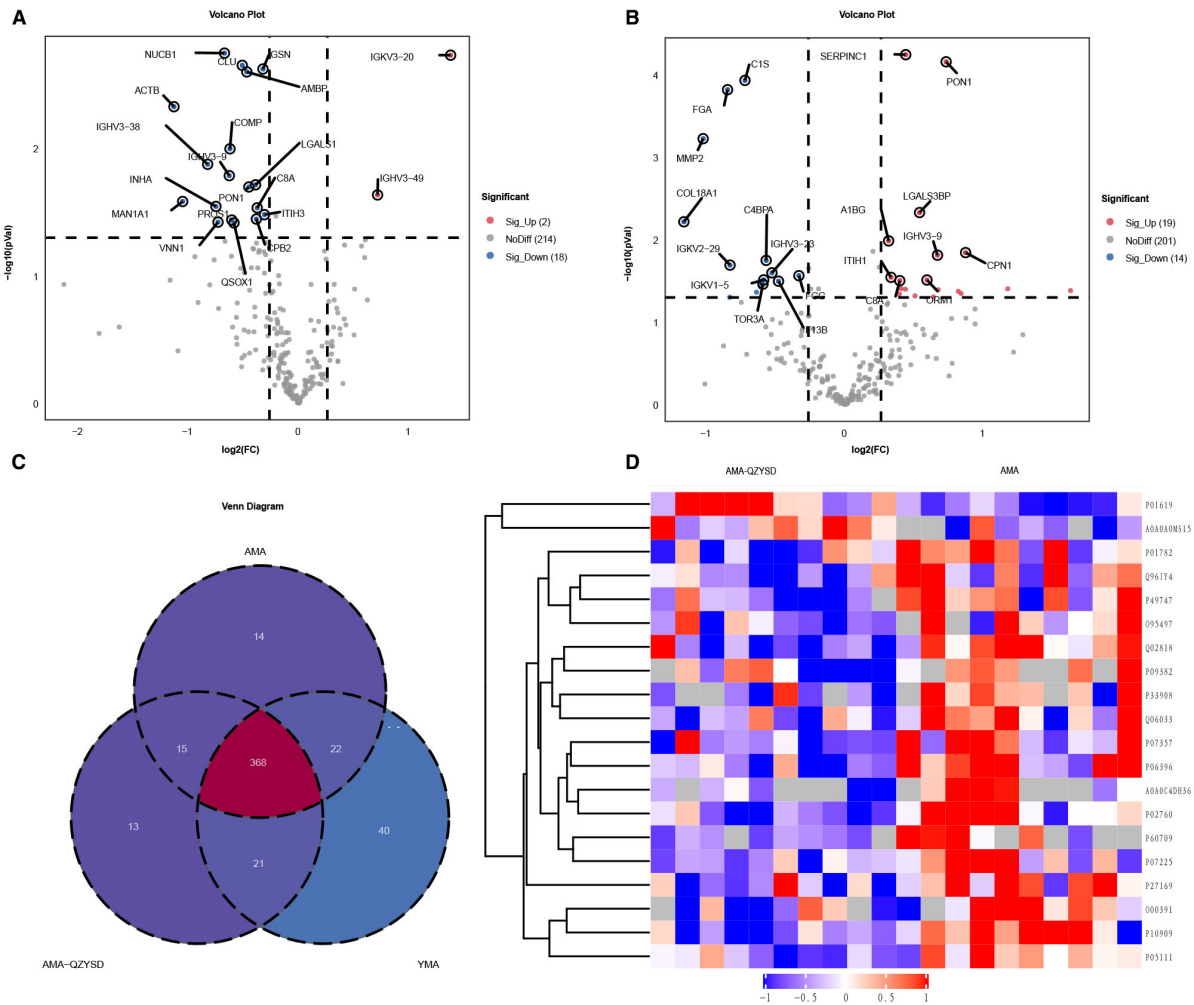
Differentially expressed proteins in follicular fluid of AMA-QZYSD vs. AMA and AMA vs. YMA. **(A)** Volcano plot of DEPs in the AMA-QZYSD and AMA control groups. **(B)** Volcano plot of DEPs in the AMA and YMA control groups. **(C)** Venn diagram showing the shared and unique identified proteins among the AMA, AMA-QZYSD, and YMA groups. **(D)** Heatmap of the expression of top 20 DEPs in the AMA-QZYSD and AMA groups. DEPs, differentially expressed proteins; FF, follicular fluid.

Significant DEPs were identified based on an expression fold change threshold of > 1.2 for upregulation and < 0.83 for downregulation, combined with a *P*-value < 0.05 (t-test or equivalent) (16). To account for multiple comparisons, FDR correction was applied, with an FDR ≤ 0.01 considered significant. When comparing AMA-QZYSD and AMA, 20 differential proteins were identified, with two upregulated (red) and 18 downregulated (blue) (**Figure 1A**, **Supplementary Table S2**). When comparing AMA and YMA, 33 differential proteins were identified, with 19 upregulated and 14 downregulated (**Figure 1B**, **Supplementary Table S3**). When comparing AMA-QZYSD versus AMA and AMA versus YMA, five significant overlapping differential proteins were identified, among which component C8 alpha chain (C8A), carboxypeptidase B2 (CPB2), serum paraoxonase/arylesterase 1 (PON1), immunoglobulin heavy variable 3-9 (IGHV3-9), pantetheinase (VNN1) exhibited opposite expression trends (*P* < 0.05) (**Table 3**). Besides, 450 proteins were identified in YMA, 416 in AMA-QZYSD, and 418 in AMA (**Figure 1C**, **Supplementary Table S4**). The cluster analysis of the heatmap also demonstrated that among these DEPs, QZYSD intervention induced significant changes in FF proteins of patients with AMA (**Figure 1D**).

**Table 3:** Differentially expressed proteins of AMA-QZYSD vs. AMA and AMA vs. YMA.

Protein ID	Protein name	Fold change	*P* value
P07357	Complement component C8 alpha chain (C8A)	0.77135248	0.029190208
Q96IY4	Carboxypeptidase B2 (CPB2)	0.767315939	0.035735379
P27169	Serum paraoxonase/arylesterase 1 (PON1)	0.731902897	0.020019765
P01782	Immunoglobulin heavy variable 3-9 (IGHV3-9)	0.647005789	0.016324212
O95497	Pantetheinase (VNN1)	0.602669823	0.037772684

*P* value, Fisher's exact test with FDR correction (FDR ≤ 0.01). AMA-QZYSD, advanced maternal age qiziyusi decoction; AMA, advanced maternal age; YMA, young maternal age.

### Bioinformatics analysis of FF proteomics results

**Figure 2:**
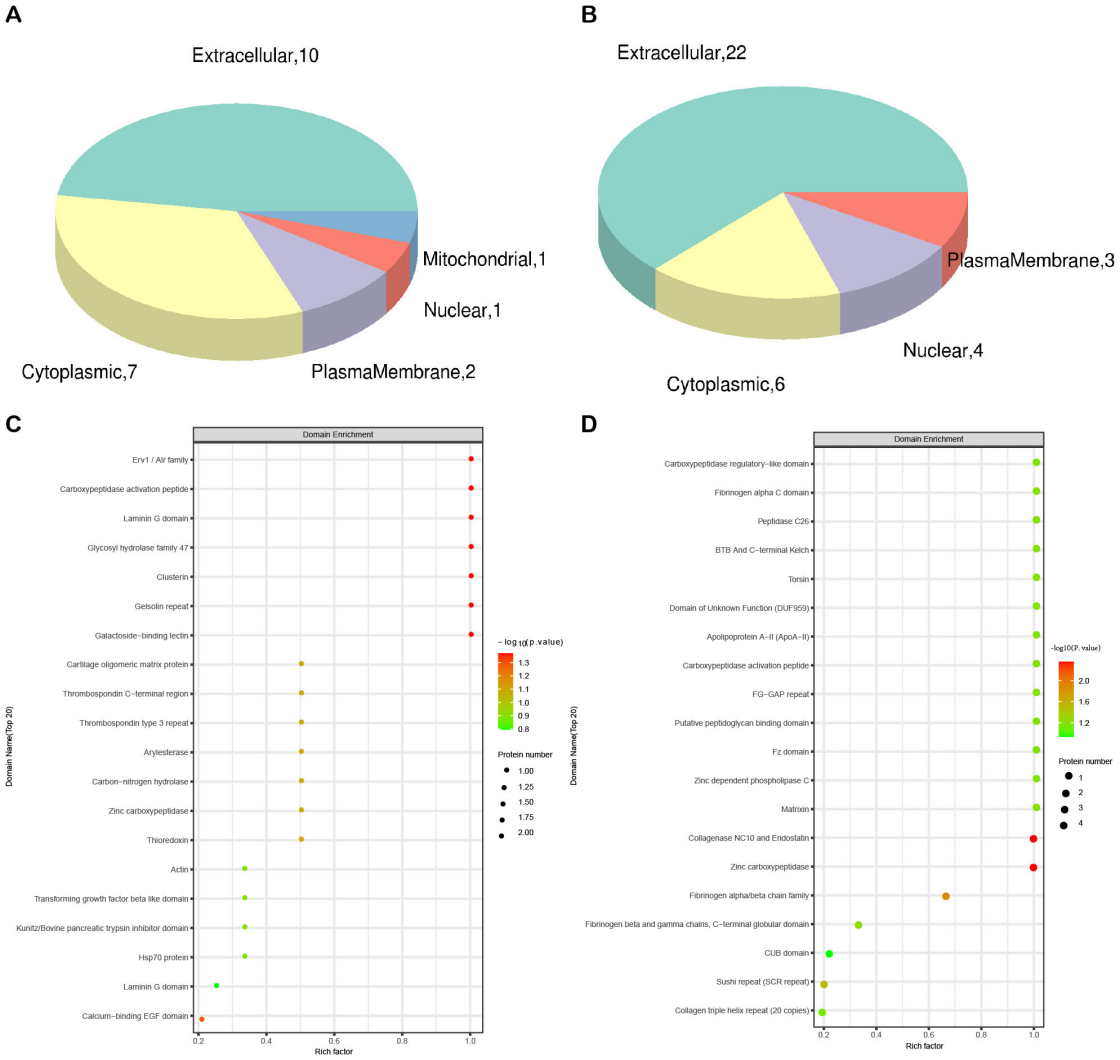
Subcellular localization and domain enrichment analysis for AMA-QZYSD vs. AMA and AMA vs. YMA. Subcellular localization analysis of AMA-QZYSD vs. AMA **(A)** and AMA vs. YMA **(B)**. Domain enrichment analysis of AMA-QZYSD vs. AMA **(C)** and AMA vs. YMA **(D)**. The x-axis represents the enrichment factor (with a maximum of 1), while the y-axis displays the statistical distribution of proteins across each domain classification. The color of the bubbles indicates the significance of enrichment for each domain, calculated using Fisher’s exact test. The color gradient (represented by -log10 of the *P*-value) shifts toward red as significance increases, meaning that a lower *P*-value corresponds to a higher significance level for enrichment in the respective domain classification.

**Figure 3:**
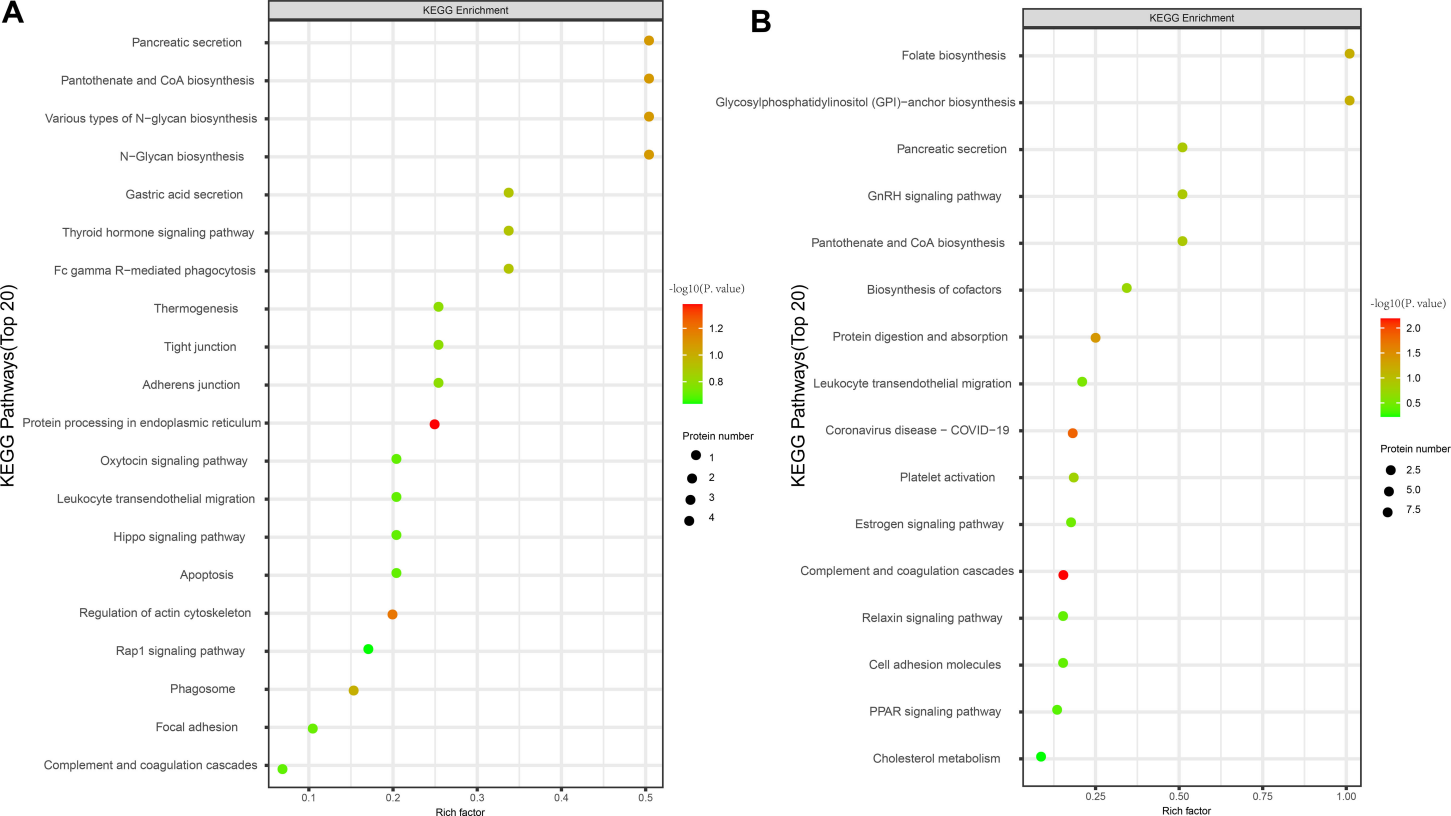
KEGG pathway enrichment analysis of differentially expressed proteins. Bubble maps of KEGG pathway enrichment of DEPs of AMA-QZYSD vs. AMA **(A)** and AMA vs. YMA **(B)**. KEGG enrichment is assessed by the Rich factor, *P*-value, and the number of genes associated with each pathway. The Rich factor represents the ratio of differentially expressed genes (DEPs) in the pathway to the total gene count within it. The P-value, ranging from 0 to 1, indicates pathway enrichment significance, with values closer to zero denoting higher significance. Bubble size corresponds to the number of DEPs meeting criteria (*P* adj < 0.05, |log2(fold change)| > 1) for KEGG enrichment. Each point signifies a KEGG pathway, with the ordinate displaying pathway names and the abscissa showing log10 (*P* value). Color intensity, from lighter to darker red, denotes increasing significance. DEPs, differentially expressed genes.

Subcellular localization analysis revealed that the differentially expressed proteins in the QZYSD-AMA versus AMA and AMA versus YMA groups were primarily localized in the extracellular and cytoplasmic compartments (**Figures 2A, B**). Domain enrichment analysis identified carboxypeptidase activation peptide and zinc carboxypeptidase as the predominant proteins in these groups (**Figures 2C, D**). The top 10 KEGG pathways enriched in the comparisons of AMA-QZYSD versus AMA and AMA versus YMA are displayed in **Table 4**. KEGG pathway enrichment analysis presented significant enrichment of the pancreatic secretion and pantothenate and coenzyme A (CoA) biosynthesis pathways in the QZYSD-AMA versus AMA and AMA versus YMA groups (**Figures 3A, B**).

**Table 4:** Top 10 KEGG pathways enriched in AMA-QZYSD vs. AMA and AMA vs. YMA.

Group	Pathway	*P* value	Impact factor
AMA-QZYSD vs. AMA	Protein processing in endoplasmic reticulum	0.04163275	0.25
	Regulation of actin cytoskeleton	0.063536967	0.2
	N-Glycan biosynthesis	0.083627242	0.5
	Various types of N-glycan biosynthesis	0.083627242	0.5
	Pantothenate and CoA biosynthesis	0.083627242	0.5
	Pancreatic secretion	0.083627242	0.5
	Phagosome	0.101951428	0.153846154
	Fc gamma R-mediated phagocytosis	0.12290036	0.333333333
	Thyroid hormone signaling pathway	0.12290036	0.333333333
	Gastric acid secretion	0.12290036	0.333333333
AMA vs. YMA	Complement and coagulation cascades	0.006469124	0.163636364
	Coronavirus disease - COVID-19	0.014624874	0.1875
	Protein digestion and absorption	0.039939008	0.25
	Glycosylphosphatidylinositol (GPI)-anchor biosynthesis	0.067073171	1
	Folate biosynthesis	0.067073171	1
	Pantothenate and CoA biosynthesis	0.129774974	0.5
	GnRH signaling pathway	0.129774974	0.5
	Pancreatic secretion	0.129774974	0.5
	Platelet activation	0.16422318	0.181818182
	Biosynthesis of cofactors	0.188381965	0.333333333

AMA-QZYSD, advanced maternal age qiziyusi decoction; YMA, young maternal age; KEGG, kyoto encyclopedia of genes and genomes.

### Metabolomic and proteomic correlation analysis

**Figure 4:**
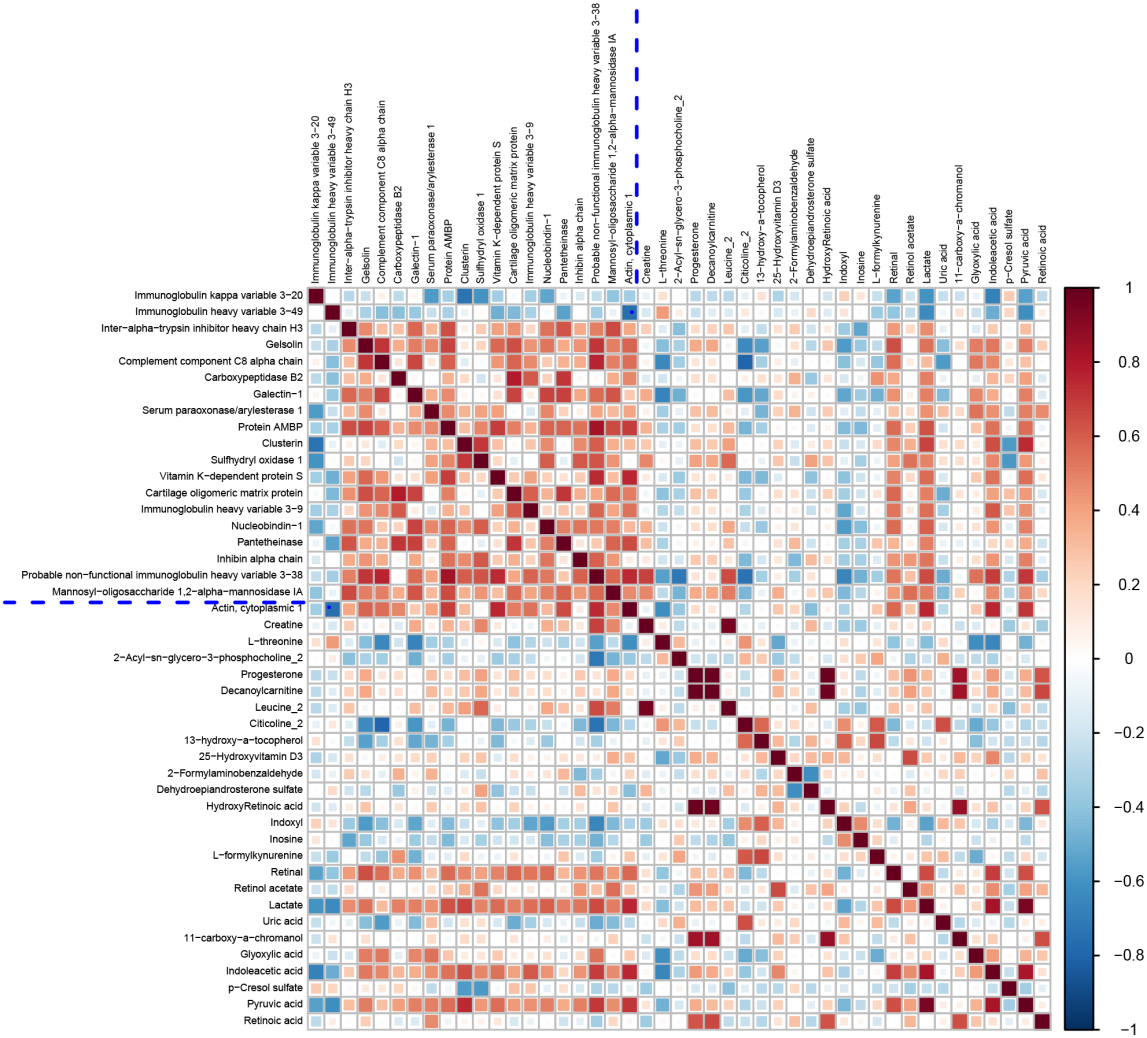
Correlation matrix heatmap of differential proteins and metabolites of AMA-QZYSD vs. AMA.This matrix plot shows the correlations between significantly differentially expressed proteins and significantly differentially expressed metabolites. The Pearson correlation coefficient (r) ranges from -1 to +1. The correlation coefficient r for proteins and metabolites is represented by color. A positive correlation (r > 0) is shown in red and a negative correlation (r < 0) is shown in blue. The deeper the color, the stronger the correlation. The blue dashed line in the figure acts as a divider; the top left quadrant displays correlations among significantly different proteins, the bottom right shows correlations among significantly different metabolites, and the top right and bottom left quadrants both show correlations between significantly different proteins and metabolites.

**Figure 5:**
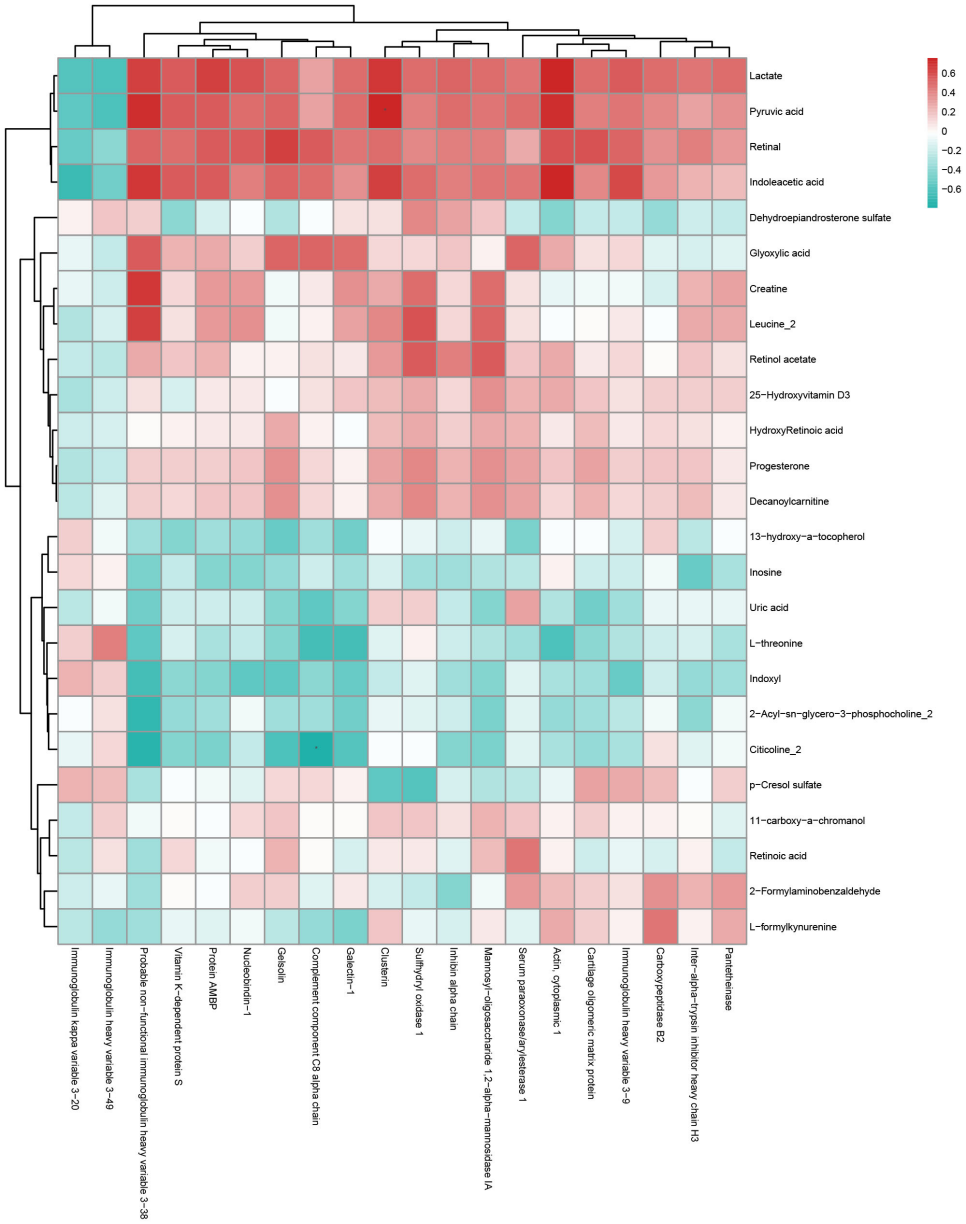
Hierarchical clustering heatmap of Pearson correlation analysis between differentially expressed proteins and metabolites of AMA-QZYSD vs. AMA. In the hierarchical clustering heatmap, each row represents a significantly different metabolite, and each column represents a significantly different protein. The dendrogram on the left represents the clustering results for differential metabolites, and the dendrogram at the top represents the clustering results for differential proteins. Significantly different metabolites or proteins clustered in the same cluster exhibit similar expression patterns. Each cell in the hierarchical clustering heatmap contains two pieces of information (correlation coefficient r and *P*-value). The correlation coefficient r is represented by color. A positive correlation (r > 0) is represented in red, while a negative correlation (r < 0) is represented in blue; the deeper the color, the stronger the correlation. The *P*-value indicates the statistical significance of the correlation.

To investigate the relationship between differential metabolites and proteins, a comprehensive assessment was conducted to explore the relationship between DEPs and DEMs in the QZYSD-AMA versus AMA and AMA versus YMA groups. The correlation matrix heatmap revealed significant associations between proteins and metabolites, with distinct clusters reflecting strong positive and negative correlations in the QZYSD-AMA versus AMA and AMA versus YMA groups (**Figures 4**, **Supplementary Figure S1**, **Table S5**). Pearson’s correlation coefficients (r) were used to quantify these relationships, with positive correlations represented in red and negative correlations in blue. The heatmap indicated that certain proteins and metabolites exhibited complementary expression patterns, while others displayed opposing trends in both the QZYSD-AMA versus AMA and AMA versus YMA groups (**Figures 5**, **Supplementary Figure S2**, **Table S5**). Notably, in both the QZYSD-AMA versus AMA and AMA versus YMA groups, the strongest correlations were observed in clusters involving key metabolic pathways, such as pancreatic secretion and pantothenate and CoA biosynthesis pathways.

**Figure 6:**
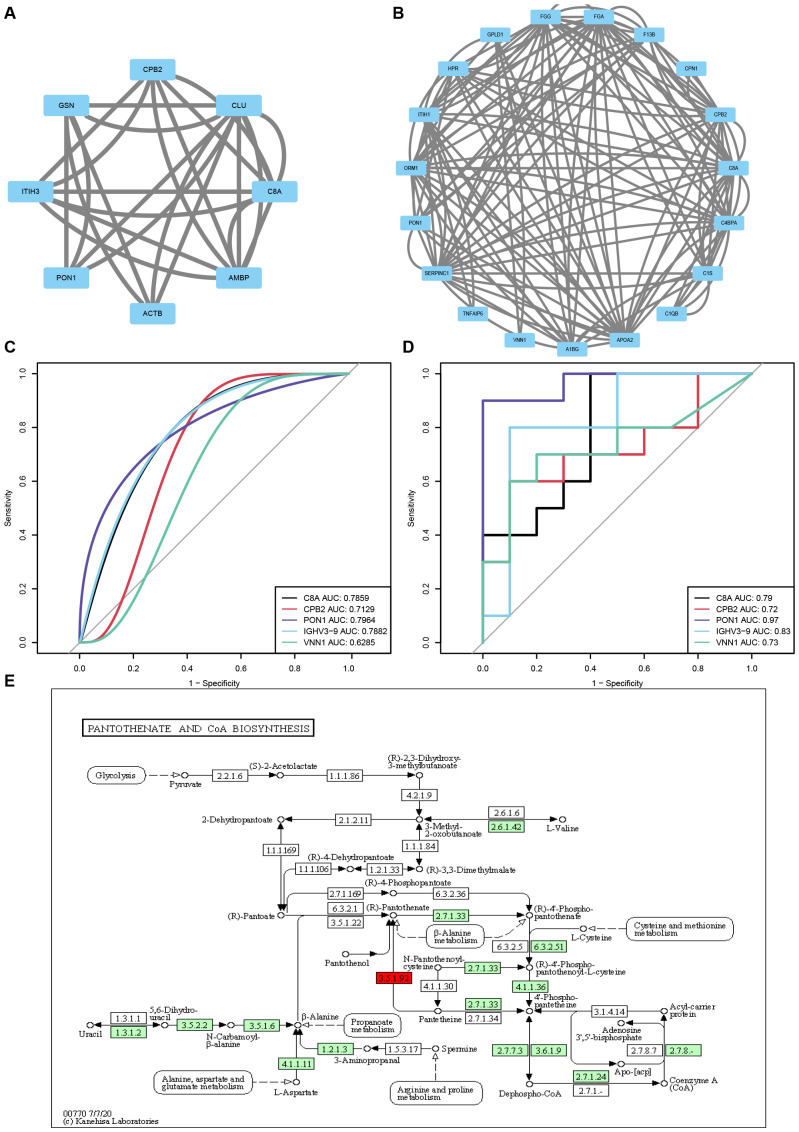
QZYSD-related core differential proteins and pathways. Interaction network diagram of DEPs for the AMA-QZYSD vs. AMA **(A)** and AMA vs. YMA **(B)**. Hub proteins identified using CytoHubba. Each circle in the figure represents a protein, and the lines between circles represent interactions between proteins. The color of a circle reflects the number of interactions that the corresponding protein has, with a darker color indicating a higher degree of connectivity. The ROC analysis results of common differential proteins C8A, CPB2, PON1, IGHV3–9 and VNN1 between AMA-QZYSD vs. AMA **(C)** and AMA vs. YMA **(D)** Differential metabolites and differential protein KEGG co-enrichment pathway. **(E)** The green background box indicates down-regulated proteins, while the yellow background box signifies the presence of both up-regulated and down-regulated proteins. Small circles represent small molecule metabolites, and large circles represent other pathways. Notably, the light green background box highlights species-specific proteins, the light purple background box also indicates species-specific proteins, and proteins labeled in red font correspond to KEGG, which is the default for disease-related proteins.

The PPI network diagram of some top DEPs is displayed in **Figures 6A, B**. Additionally, an ROC analysis was performed to evaluate the sensitivity and specificity of the core proteins in detecting the improvement of reproductive outcomes in AMA infertility by QZYSD. The area under the curve (AUC) values for five proteins depicted that PON1 had AUC values of 0.97 and 0.80 in predicting AMA infertility and the efficacy of TCM, respectively (**Figures 6C, D**). These results suggest that PON1 and the pantothenate and CoA biosynthesis pathways may serve as potential targets for addressing AMA infertility and the effects of the QZYSD intervention (**Figure 6E**). These findings highlight the intricate interplay between proteomic and metabolomic profiles and provide insights into the molecular mechanisms underlying the observed physiological changes.

## Discussion

As life expectancy and living standards have increased, the age of childbearing in women has been gradually delayed. AMA has become among the main causes of infertility, primarily leading to a decline in the quantity and quality of oocytes, along with an increased risk of obstetric complications and offspring health issues (3, 17). Although assisted reproductive technology (ART) is the primary approach for treating infertility in older women, challenges remain, including a decline in ART success rates with increasing age (6, 18). TCM, with its long history and proven therapeutic effects, has been widely accepted and applied in the treatment of various clinical conditions, particularly gynecological disorders, such as AMA infertility. In this context, we first conducted a prospective cohort study to assess the role of TCM in improving infertility linked to advanced AMA. Additionally, we employed a combined analysis of proteomics and metabolomics to identify potential FF biomarkers and mechanisms through which TCM acts. We hope this study will provide valuable insights into the biomarkers of oocyte quality and quantity decline and how TCM may improve the treatment of AMA infertility, offering more effective clinical strategies.

TCM has unique advantages in treating gynecological conditions, particularly in regulating menstrual cycles and supporting pregnancy, and it plays a vital role in maintaining health and reproductive function. QZYSD, a routinely used at our institution of the Integrated Reproductive Medicine Center of this hospital, has demonstrated significant clinical efficacy in assisting women with AMA undergoing ART (8). Our study further confirms that QZYSD can significantly improve the number of retrieved oocytes, the number of high-quality embryos on day 3 in women with AMA undergoing ART treatment, which aligns with previous findings (8) Although the CCP rate in the AMA-QZYSD group did not exhibit a statistically significant difference when compared to the AMA group, as reported by Zhang et al. (8), a discernible trend towards a higher CCP rate in the AMA-QZYSD group was observed. This discrepancy may be attributed to variations in research methodologies between the two studies, as well as the relatively small sample size in our study. A key factor in oocyte quality is FF, which plays a crucial role in oocyte growth and maturation by providing essential nutrients and signals. Consequently, through multi-omics analysis of FF, we identified potential biomarkers and targets related to AMA infertility and the effects of QZYSD, thereby providing new directions for clinical application.

C8A, an essential molecule in immune response, may influence oocyte development and quality by modulating the immune response in the follicular microenvironment (19). Previous studies have exhibited that immune system dysregulation can affect oocyte maturation and its interaction with follicles, ultimately affecting fertilization rates (20, 21). Previous studies have revealed that CPB2 has anti-inflammatory effects (22). Its expression is downregulated in the FF of women with AMA infertility. In contrast, QZYSD intervention increases its expression, suggesting its role in both the pathogenesis and intervention of AMA infertility.

PON1, an antioxidant enzyme, is involved in inflammation, oxidative stress, and lipid metabolism (23), key factors influencing oocyte quality and quantity in women with AMA. A study of the ovarian stimulation cycle in women revealed that follicular fluid PON1 arylesterase and paraoxonase activity was positively correlated with the number of retrieved oocytes (24). Human ovarian granulosa cells express the genes PON1 may be synthesized by ovarian cells, and PON1 expression and subcellular distribution was associated with the cell cycle (25). PON1 enhances the developmental rate of bovine embryos during *in vitro* maturation, promoting progression to the blastocyst stage (26).

Moreover, IGHV3-9, related to immune responses and metabolic processes (27), may affect oocyte physiology and follicle development, particularly in elderly individuals, where metabolic imbalances contribute to ovarian aging and reduced fertility. Finally, due to its ability to influence multiple metabolic pathways and modulate oxidative stress, VNN1 has become a critical factor in the progression of various diseases (28). Animal studies have also revealed that VNN1 is involved in movento-mediated ovarian granulosa cell dysfunction and follicular developmental disorders (29). CoA and acetyl-CoA are key regulators of cellular energy metabolism and play central roles in the production and breakdown of major energy sources in the body (30, 31). Through a comprehensive analysis, we identified that the CoA pathway plays a crucial role in both AMA infertility and the therapeutic effects of QZYSD, aligning with the high metabolic and energy demands of oocytes. This suggests that the A pathway may play a significant role in the pathogenesis of AMA infertility.

This study also has several notable limitations that should be addressed in future research. First, the evaluation of QZYSD’s effect on improving infertility in older women was not conducted through a randomized, placebo controlled, double-blind trial, and there was no statistical analysis of outcomes related to fresh and frozen embryo transfer cycles and rates of on day 5 blastocyst and live birth. Second, while the study integrated metabolomics and proteomics to explore the mechanisms by which QZYSD improves infertility, the large dataset made it challenging to analyze each result individually. Consequently, only correlation analysis, focusing on predicting metabolic pathways, was conducted to infer the mechanisms of QZYSD. Third, due to the involvement of multiple pathways, a comprehensive comparison of all enriched pathways was not performed, and further validation and analysis were not performed. Our differential-proteomics filter used a relatively lenient fold-change threshold, intentionally retaining modest effect sizes that are common for cytokines and secreted factors. This choice prioritizes sensitivity over specificity and may increase the risk of false-positive candidates. Accordingly, we present full effect-size estimates and statistics for transparency, interpret these findings as hypothesis-generating, and emphasize the need for orthogonal validation (e.g., PRM/SRM-based targeted MS, ELISA or immunoblotting) and replication in larger cohorts. We also complement single-protein results with pathway-level analyses to reduce noise at the individual-feature level; however, such enrichment analyses do not substitute for experimental validation. Future work will evaluate stricter fold-change cut-offs in confirmatory datasets to assess the robustness of the observed patterns. Finally, the study lacked gene knockout or *in vitro* cell-based validation experiments, essential for further elucidating the mechanisms by which QZYSD enhances fertility in older women.

In conclusion, our study provides robust evidence supporting the role of QZYSD in improving ART outcomes in women with AMA, likely through its effects on key metabolic and proteomic pathways in FF. Given that differentially expressed proteins and pathways are involved in inflammatory immune responses and oxidative stress, this also indirectly suggests that the decline in oocyte quantity and quality due to aging is an inflammation-driven event. By identifying biomarkers and molecular targets related to oocyte quality and ART success, we enhance our understanding of how TCM can be integrated into modern ART practices. This study lays the foundation for future research to explore the full potential of QZYSD in improving reproductive health for aging women and broader fertility management. Further studies with larger sample sizes, extended follow-up periods, and advanced analytical techniques are required to fully elucidate the long-term benefits and mechanisms of QZYSD. Ultimately, these efforts will contribute to the development of more effective and personalized treatments for infertility in women with AMA.

## Data Availability

The original contributions presented in the study are included in the article/**Supplementary Material**. Further inquiries can be directed to the corresponding author/s.

## Supplementary material

Supplementary Figure 1 Correlation Matrix Heatmap of Significantly Differential Proteins and Metabolites of AMA vs. YMA. This matrix plot shows the correlations between significantly differentially expressed proteins and significantly differentially expressed metabolites. The Pearson correlation coefficient (r) ranges from -1 to +1. The correlation coefficient r for proteins and metabolites is represented by color. A positive correlation (r > 0) is shown in red and a negative correlation (r < 0) is shown in blue. The deeper the color, the stronger the correlation. The blue dashed line in the figure acts as a divider; the top left quadrant displays correlations among significantly different proteins, the bottom right shows correlations among significantly different metabolites, and the top right and bottom left quadrants both show correlations between significantly different proteins and metabolites.

Supplementary Figure 2 Hierarchical clustering heatmap of Pearson correlation analysis between differentially expressed proteins and metabolites of AMA vs. YMA. In the hierarchical clustering heatmap, each row represents a significantly different metabolite, and each column represents a significantly different protein. The dendrogram on the left represents the clustering results for differential metabolites, and the dendrogram at the top represents the clustering results for differential proteins. Significantly different metabolites or proteins clustered in the same cluster exhibit similar expression patterns. Each cell in the hierarchical clustering heatmap contains two pieces of information (correlation coefficient r and *P*-value). The correlation coefficient r is represented by color. A positive correlation (r > 0) is represented in red, while a negative correlation (r < 0) is represented in blue; the deeper the color, the stronger the correlation. The *P*-value indicates the statistical significance of the correlation.

Supplementary Table 1 Parameters and protocols of protein extraction and LC-MS/MS analysis.

Supplementary Table 2 Differentially expressed proteins of AMA-QZYSD vs. AMA.

Supplementary Table 3 Differentially expressed proteins of AMA vs. YMA.

Supplementary Table 4 Overview of protein identification and quantification.

Supplementary Table 5 Correlation network of differential proteins and metabolites.
