# Supplementary figures and images for: Integrated proteomic and metabolomic analysis elucidates the effects and mechanisms of Qiziyusi decoction on IVF outcomes in advanced maternal age infertility
Source: Front Endocrinol (Lausanne). 2025 Oct 10;16:1573206. doi: 10.3389/fendo.2025.1573206 (PMC12549270; doi:10.3389/fendo.2025.1573206)

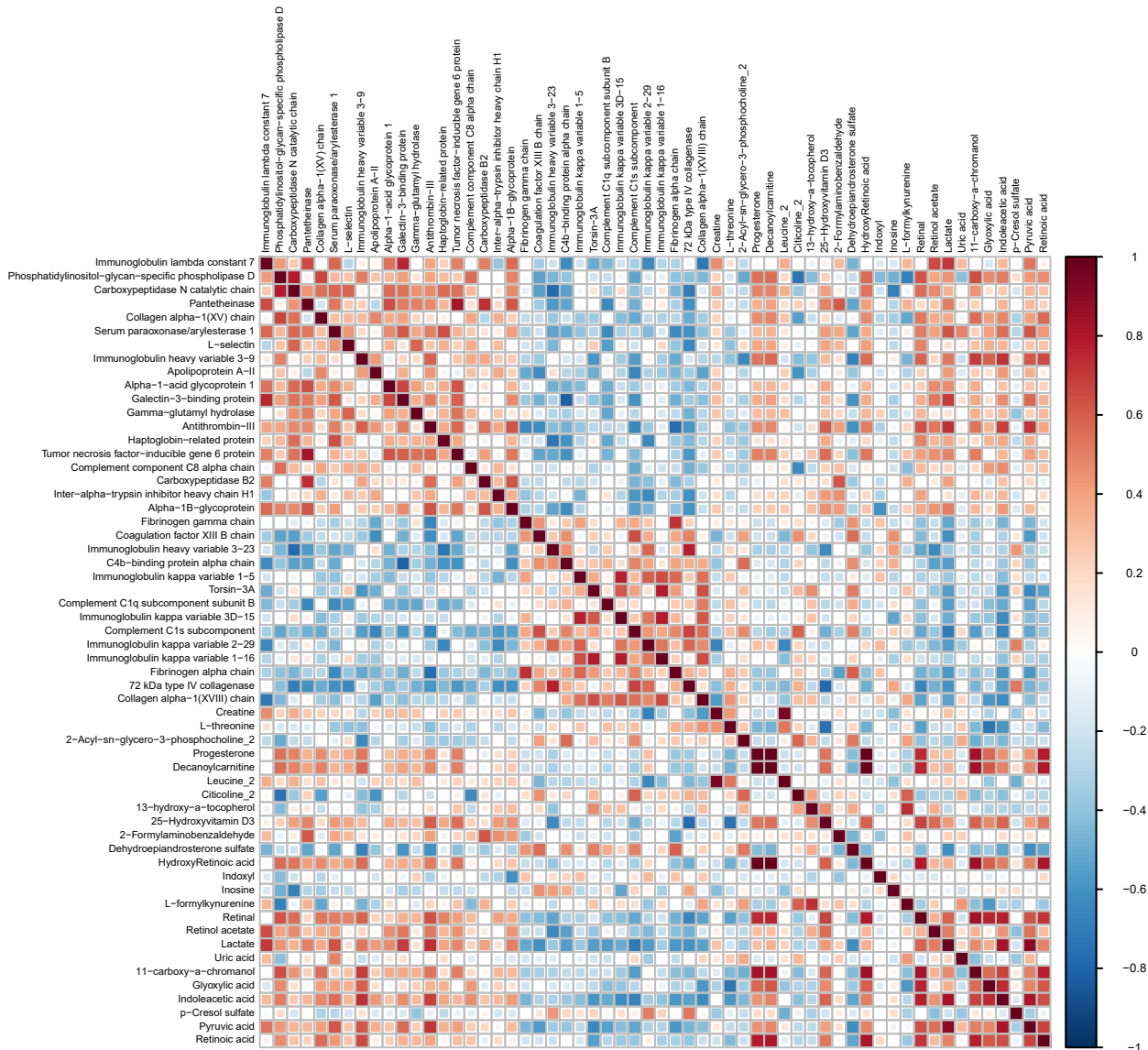

Supplement: Supplementary Figure 1 — Correlation Matrix Heatmap of Significantly Differential Proteins and Metabolites of AMA vs. YMA. This matrix plot shows the correlations between significantly differentially expressed proteins and significantly differentially expressed metabolites. The Pearson correlation coefficient (r) ranges from -1 to +1. The correlation coefficient r for proteins and metabolites is represented by color. A positive correlation (r > 0) is shown in red and a negative correlation (r < 0) is shown in blue. The deeper the color, the stronger the correlation. The blue dashed line in the figure acts as a divider; the top left quadrant displays correlations among significantly different proteins, the bottom right shows correlations among significantly different metabolites, and the top right and bottom left quadrants both show correlations between significantly different proteins and metabolites. [file DataSheet1.pdf]

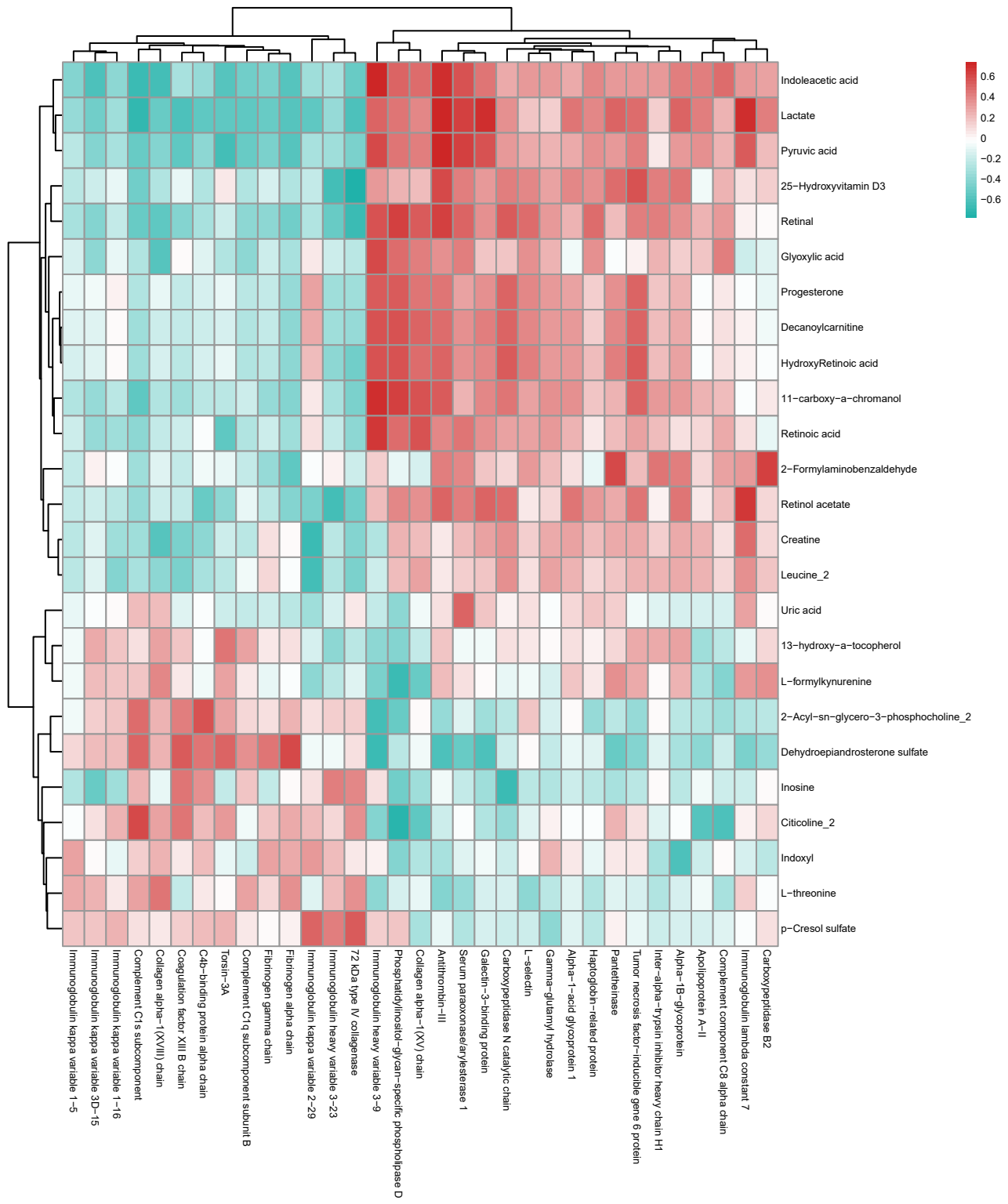

Supplement: Supplementary Figure 2 — Hierarchical clustering heatmap of Pearson correlation analysis between differentially expressed proteins and metabolites of AMA vs. YMA. In the hierarchical clustering heatmap, each row represents a significantly different metabolite, and each column represents a significantly different protein. The dendrogram on the left represents the clustering results for differential metabolites, and the dendrogram at the top represents the clustering results for differential proteins. Significantly different metabolites or proteins clustered in the same cluster exhibit similar expression patterns. Each cell in the hierarchical clustering heatmap contains two pieces of information (correlation coefficient r and P-value). The correlation coefficient r is represented by color. A positive correlation (r > 0) is represented in red, while a negative correlation (r < 0) is represented in blue; the deeper the color, the stronger the correlation. The P-value indicates the statistical significance of the correlation. [file DataSheet2.pdf]
