# Supplementary material for: Integrated proteomic and metabolomic analysis elucidates the effects and mechanisms of Qiziyusi decoction on IVF outcomes in advanced maternal age infertility
Source: Front Endocrinol (Lausanne). 2025 Oct 10;16:1573206. doi: 10.3389/fendo.2025.1573206 (PMC12549270; doi:10.3389/fendo.2025.1573206)
Supplement: Supplementary Table 1 — Parameters and protocols of protein extraction and LC-MS/MS analysis. [file Table1.docx]

Table S1. Parameters and protocols of protein extraction and LC-MS/MS analysis

| Category | Item | Detailed Parameters/Procedure | Reagents/Equipment |
| --- | --- | --- | --- |
| Protocol | Protein Extraction | 1. Add SDT buffer  2. Sonicate: 80 W, 10 s on/15 s off ×10 cycles.  3. Boil at 100°C for 15 min.  4. Centrifuge at 14,000 g for 40 min.  5. Aliquot and store at −80°C.  6. Mix 20 µg protein with 5× loading buffer, boil at 100°C for 5 min.  7. 12.5% SDS-PAGE electrophoresis: constant current of 14 mA for 90 min.  8. Coomassie Brilliant Blue | 1. SDT buffer: 4% SDS，100 mM Tris‐HCl，1 mM DTT，pH 7.6  2. 5× loading buffer: 10% SDS, 0.5% bromophenol blue, 50% glycerol, 500 mM DTT, 250 mM Tris-HCl, pH 6.8 |
|  | Trypsin Digestion | 1. Add DTT to 30 μL of protein solution (final 100 mM), heat at 95°C for 15 min, cool to room temperature.  2. Add 200 μL UA buffer, transfer to a 10 kD ultrafiltration centrifuge tube at 14,000 g for 15 min, discard the filtrate and repeat.  3. Add 100μL IAA buffer（100mM IAA in UA）, shake at 600 rpm for 1 minute, dark for 30 min, centrifuge at 14,000 g for 15 min, repeat twice.  4. Add 100 μL 25 mM NH4HCO3, centrifuge at 14,000 g for 40 min, repeat twice.  5. Add 40 μL Trypsin buffer（4 μg Trypsin in 40 μL 100 mM NH4HCO3）, shake at 600 rpm for 1 min, 37 °C for 16-18 h.  6. Replace the collection tube, centrifuge at 14,000 g for 15 min, add 40 μL 25 mM NH4HCO3, centrifuge at 14,000 g for 15 min, collect the filtrate.  7. Digested peptides were desalted using C18 cartridges and then add 40 μL of 0.1% formic acid after peptide segment freeze-drying. | 1. Dithiothreitol: DTT, 161‐0404, Bio-Rad)  2. UA buffer：8 M urea，150 mM Tris‐HCl，pH 8.0  3. Iodoacetamide: IAA, 163‐2109 Bio-Rad)  4. NH4HCO3: A6141 Sigma  5. Trypsin: 317107 Promega  6. C18 Cartridge: 66872‐U Sigma  7. Formic acid: FA,06450Fluka |
| LC-MS/MS analysis | NanoLC System  (Easy nLC, Thermo) | 1. Columns:  • Loading column: Thermo Scientific Acclaim PepMap 100, 100 μm×2 cm, nanoViper C18  • Analytical column: Thermo Scientific EASY column, 10 cm, ID 75μm, 3μm, C18-A2  2. Flow rate: 300 nL/min |  |
|  | Mass Spectrometer  (timsTOF Pro) | 1. Lon source voltage: 1.5 kV (positive ions)  2. Scanning range: 100-1700 m/z  3. PASEF mode: 10 MS/MS/cycle (1.17 s/cycle, 0-5 charges range)  4. Dynamic exclusion: 24 s | 1. Phase A: 0.1% acetic acid aqueous solution  2. Phase B: 0.1% formic acid acetonitrile solution (84% acetonitrile) |
