# Supplementary material for: Integrated proteomic and metabolomic analysis elucidates the effects and mechanisms of Qiziyusi decoction on IVF outcomes in advanced maternal age infertility
Source: Front Endocrinol (Lausanne). 2025 Oct 10;16:1573206. doi: 10.3389/fendo.2025.1573206 (PMC12549270; doi:10.3389/fendo.2025.1573206)
Supplement: Supplementary Table 2 — Differentially expressed proteins of AMA-QZYSD vs. AMA. [file Table2.docx]

| Tbale S2. Differentially expressed proteins of AMA-QZYSD vs. AMA | | | |
| --- | --- | --- | --- |
| Protein ID | Protein Name | Fold Change | *P* value |
| P01619 | Immunoglobulin kappa variable 3-20 (IGKV3-20) | 2.609991547 | 0.001854692 |
| A0A0A0MS15 | Immunoglobulin heavy variable 3-49 (IGHV3-49) | 1.64681611 | 0.023115216 |
| Q06033 | Inter-alpha-trypsin inhibitor heavy chain H3 (ITIH3) | 0.807635883 | 0.033010617 |
| P06396 | Gelsolin GSN() | 0.80006563 | 0.002378469 |
| P07357 | Complement component C8 alpha chain (C8A) | 0.77135248 | 0.029190208 |
| Q96IY4 | Carboxypeptidase B2 (CPB2) | 0.767315939 | 0.035735379 |
| P09382 | Galectin-1 (LGALS1) | 0.763545666 | 0.019258914 |
| P27169 | Serum paraoxonase/arylesterase 1 (PON1) | 0.731902897 | 0.020019765 |
| P02760 | Protein AMBP (AMBP) | 0.722561882 | 0.002507579 |
| P10909 | Clusterin (CLU) | 0.70212315 | 0.002227225 |
| O00391 | Sulfhydryl oxidase 1 (QSOX1) | 0.666309667 | 0.038283486 |
| P07225 | Vitamin K-dependent protein S (PROS1) | 0.657324269 | 0.036363394 |
| P49747 | Cartilage oligomeric matrix protein (COMP) | 0.649840621 | 0.010056792 |
| P01782 | Immunoglobulin heavy variable 3-9 (IGHV3-9) | 0.647005789 | 0.016324212 |
| Q02818 | Nucleobindin-1 (NUCB1) | 0.62780039 | 0.001787247 |
| O95497 | Pantetheinase (VNN1) | 0.602669823 | 0.037772684 |
| P05111 | Inhibin alpha chain (INHA) | 0.594819376 | 0.028442031 |
| A0A0C4DH36 | Probable non-functional immunoglobulin heavy variable 3-38 (IGHV3-38) | 0.564624972 | 0.013301365 |
| P33908 | Mannosyl-oligosaccharide 1,2-alpha-mannosidase IA (MAN1A1) | 0.482351296 | 0.02600963 |
| P60709 | Actin, cytoplasmic 1 (ACTB) | 0.456581616 | 0.004701055 |

*P* value, Fisher's exact test with FDR correction (FDR ≤ 0.01). AMA-QZYSD, advanced maternal age qiziyusi decoction; YMA, young maternal age.
