# Supplementary material for: Integrated proteomic and metabolomic analysis elucidates the effects and mechanisms of Qiziyusi decoction on IVF outcomes in advanced maternal age infertility
Source: Front Endocrinol (Lausanne). 2025 Oct 10;16:1573206. doi: 10.3389/fendo.2025.1573206 (PMC12549270; doi:10.3389/fendo.2025.1573206)
Supplement: Supplementary Table 3 — Differentially expressed proteins of AMA vs. YMA. [file Table3.docx]

| Table S3. Differentially expressed proteins of AMA vs. YMA | | | |
| --- | --- | --- | --- |
| Protein ID | Protein Name | Fold Change | *P*-value |
| A0M8Q6 | Immunoglobulin lambda constant 7 (IGLC7) | 3.108427861 | 0.041288117 |
| P80108 | Phosphatidylinositol-glycan-specific phospholipase D (GPLD1) | 2.267783992 | 0.039210465 |
| P15169 | Carboxypeptidase N catalytic chain (CPN1) | 1.834796452 | 0.014265531 |
| O95497 | Pantetheinase (VNN1) | 1.794112437 | 0.044790838 |
| P39059 | Collagen alpha-1(XV) chain (COL15A1) | 1.769127715 | 0.042103312 |
| P27169 | Serum paraoxonase/arylesterase 1 (PON1) | 1.664043311 | 6.90455E-05 |
| P14151 | L-selectin (SELL) | 1.599015503 | 0.040199273 |
| P01782 | Immunoglobulin heavy variable 3-9 (IGHV3-9) | 1.593258238 | 0.015341396 |
| P02652 | Apolipoprotein A-II (APOA2) | 1.561207661 | 0.049318427 |
| P02763 | Alpha-1-acid glycoprotein 1 (ORM1) | 1.511345315 | 0.030678923 |
| Q08380 | Galectin-3-binding protein (LGALS3BP) | 1.456038091 | 0.004659065 |
| Q92820 | Gamma-glutamyl hydrolase (GGH) | 1.422893204 | 0.047809617 |
| P01008 | Antithrombin-III (SERPINC1) | 1.358430174 | 5.66072E-05 |
| P00739 | Haptoglobin-related protein (HPR) | 1.356671459 | 0.039746158 |
| P98066 | Tumor necrosis factor-inducible gene 6 protein (TNFAIP6) | 1.326257941 | 0.039036058 |
| P07357 | Complement component C8 alpha chain (C8A) | 1.318542085 | 0.031258419 |
| Q96IY4 | Carboxypeptidase B2 (CPB2) | 1.315446816 | 0.045471143 |
| P19827 | Inter-alpha-trypsin inhibitor heavy chain H1 (ITIH1) | 1.261771193 | 0.02847845 |
| P04217 | Alpha-1B-glycoprotein (A1BG) | 1.246743105 | 0.010280648 |
| P02679 | Fibrinogen gamma chain (FGG) | 0.794495129 | 0.02720146 |
| P05160 | Coagulation factor XIII B chain (F13B) | 0.717130936 | 0.031741522 |
| P01764 | Immunoglobulin heavy variable 3-23 (IGHV3-23) | 0.693816692 | 0.025113511 |
| P04003 | C4b-binding protein alpha chain (C4BPA) | 0.674032082 | 0.017741289 |
| P01602 | Immunoglobulin kappa variable 1-5 (IGKV1-5) | 0.665737331 | 0.030592029 |
| Q9H497 | Torsin-3A (TOR3A) | 0.663208954 | 0.034434657 |
| P02746 | Complement C1q subcomponent subunit B (C1QB) | 0.655221066 | 0.037076046 |
| A0A087WSY6 | Immunoglobulin kappa variable 3D-15 (IGKV3D-15) | 0.64218658 | 0.043102246 |
| P09871 | Complement C1s subcomponent (C1S) | 0.606005382 | 0.000116102 |
| A2NJV5 | Immunoglobulin kappa variable 2-29 (IGKV2-29) | 0.562208161 | 0.020243995 |
| P04430 | Immunoglobulin kappa variable 1-16 (IGKV1-16) | 0.561358203 | 0.04964628 |
| P02671 | Fibrinogen alpha chain (FGA) | 0.555284823 | 0.000150595 |
| P08253 | 72 kDa type IV collagenase (MMP2) | 0.491119032 | 0.000591945 |
| P39060 | Collagen alpha-1(XVIII) chain (COL18A1) | 0.445875252 | 0.006034673 |

*P* value, Fisher's exact test with FDR correction (FDR ≤ 0.01). AMA, advanced maternal age; YMA, young maternal age.
